# Case of Poisoning by Laudanum

**Published:** 1851-08

**Authors:** 


*Case of Poisoning by Laudanum, resulting from a Mistake in a Physician's Prescription.*—A case which has caused much sensation among the profession at Paris, was lately tried before the tribunal correctionel. The facts are briefly these:—In October, 1850, a Mr. Labbé, post-master at Alfort, near Paris, was attended for a severe indisposition, by Dr. Deguise, a highly respected practitioner of the French capital. In the course of the treatment, Dr. Deguise prescribed an injection with laudanum, and instead of writing *ten drops*, wrote *ten grammes.* (The gramme is equivalent to about fifteen grains.) The patient was soon afterwards thrown into a comatose state; but by proper treatment was completely roused, and remained quite conscious, and free from narcotic symptoms, for twenty hours. After this period he, however, suddenly grew worse, and died two days after the injection had been administered.

The authorities appointed th ee commissioners, Messrs. Cruveilheir, Devergie, and Rayer, to investigate the matter, the result of the inquiry was, that the commissioners were of opinion, that the patient had died of a low fever, *complicated* by an overdose of laudanum. The court, not seeing in this answer whether the death had resulted from the imprudence of the medical attendant or not, disregarded the verdict of the commissioners, (without ordering a post-mortem examination,) and put Dr. Deguise on his trial. The most eminent men of the faculty were examined, among whom was Orfila, the points of interest being the quantity of laudanum injected, and the period (twenty hours) during which lucidity had been regained. The medical testimony was mainly in support of the commissioners' opinion, but the court found the accused guilty of *homicide by imprudence*, and fined him four pounds, with costs. The laudanum used was the preparation which goes by the name of Sydenham's laudanum, or compound opium-wine of the Parisian codex. We shall just transcribe its composition: Opium, two ounces: cinnamon and cloves, of each, one drachm: malaga wine, one pound. Twenty drops contain one grain of opium.—*Lon. Lancet.*

